# Stratification of atopic dermatitis patients by patterns of response to proactive therapy with topical tacrolimus: low serum IgE levels and inadequately controlled disease activity at the start of treatment predict its failure

**DOI:** 10.1080/07853890.2021.2004319

**Published:** 2021-11-19

**Authors:** Hiroko Kasai, Hiroshi Kawasaki, Ayano Fukushima-Nomura, Fumiyo Yasuda-Sekiguchi, Masayuki Amagai, Tamotsu Ebihara, Keiji Tanese

**Affiliations:** a Department of Dermatology, Keio University School of Medicine, Tokyo, Japan; b Department of Dermatology, Kitasato University Kitasato Institute Hospital, Tokyo, Japan; c Medical Sciences Innovation Hub Program, RIKEN, Kanagawa, Japan; d Department of Dermatology, Tokyo Saiseikai Central Hospital, Tokyo, Japan

**Keywords:** Atopic dermatitis, IgE, proactive therapy, tacrolimus, TARC

## Abstract

**Purpose:**

Topical calcineurin inhibitors (TCIs) are an important anti-inflammatory drug for treating atopic dermatitis (AD). However, those treatment responses are variable. In this study, we stratified AD patients by patterns of response to remission maintenance therapy (proactive therapy) with topical tacrolimus, a typical TCI. Thereafter, we explored patient features that predict the success or failure of proactive therapy using TCI (TCI proactive therapy).

**Methods:**

A single-arm open-label clinical study aimed to evaluate the efficacy of TCI proactive therapy was conducted in 31 patients with AD. Patients were treated with TCS to induce remission (remission-induction period) followed by daily TCI ointment (0.1% tacrolimus) application for 4 weeks (maintenance therapy period), and twice-weekly application for 12 weeks (proactive therapy period). Based on its results, treatment outcomes were correlated with the patients’ clinical and laboratory findings.

**Results:**

Of the 31 patients enrolled in the study, 21 successfully completed maintenance therapy (TCI responders). Among them, 13 completed (proactive-completed group) and 8 failed proactive therapy (proactive-dropout group). At the beginning of maintenance therapy, the serum IgE level was significantly higher in the TCI responders than in those who failed maintenance therapy (*p* = 0.049). At the beginning of proactive therapy, the mean-SCORing Atopic Dermatitis (SCORAD) score was significantly different between the proactive-completed (11.7 ± 4.6) and proactive-dropout (16.6 ± 4.2) groups (*p* = 0.025). In proactive-dropout group patients, worsened disease activity correlated well with the elevation of serum lactate dehydrogenase (LDH) and Thymus and activation-regulated chemokine (TARC) levels and peripheral eosinophil count.

**Conclusion:**

AD patients were stratified into three different response patterns to TCI proactive therapy. Patients with less involvement of IgE in the pathogenesis and inadequate remission induction by TCS may not be expected to respond well to TCI proactive therapy.

**Key messages**
- AD patients can be stratified into three types according to their pattern of responsiveness to TCI proactive therapy.
- The efficacy of TCI proactive therapy is lower in AD patients with lower serum IgE levels.
- TCI proactive therapy should be done after the achievement of adequate remission induction by TCS.

## Introduction

Atopic dermatitis (AD) is a chronic relapsing inflammatory skin disease characterised by dry skin, eczematous lesions, and severe pruritus [1]. Its aetiology is multifactorial and includes impaired skin barrier function due to filaggrin mutations, immune abnormalities such as increased Th2 immune responses, and alterations in the skin's resident flora [2].

The goals of AD treatment are to reduce skin inflammation and pruritis, prevent exacerbations, restore skin barrier function, and minimise therapeutic risks [3]. The standard treatment approach is the use of topical anti-inflammatory agents along with skin moisturisers. The U.S. Food and Drug Administration (FDA) currently approves the use of corticosteroids, calcineurin inhibitors, and phosphodiesterase-4 inhibitors [2]. They can be used reactively, (e.g. *via* daily application on active eczematous lesions) or proactively, with long-term, intermittent application (e.g. twice weekly) to maintain remission for the previously affected areas with subclinical inflammation [4,5]. Of these agents, topical corticosteroids (TCSs) are the therapeutic mainstay [6]. However, long-term TCS use can cause multiple side effects on the skin, such as acneiform eruptions, skin atrophy, and telangiectasia.

To minimise the side effects of TCS, topical calcineurin inhibitors (TCIs) is widely used as an alternative therapy for more than 20 years [7]. TCIs bind to intracellular FK-506 binding protein 12, forming a complex that inhibits calcineurin phosphatase activity, thereby blocking T-cell activation [8]. TCIs also inhibit epidermal antigen-presenting dendritic cells, reducing the immune response to antigens [7]. The FDA has currently approved the use of two TCIs, tacrolimus and pimecrolimus [3,4]. TCIs have the same potency as moderate-strength TCSs and are used to treat mild to moderate eczema lesions [4]. In AD patients with recurrent eczema, proactive therapy with TCIs (TCI proactive therapy) has been recommended as an effective management therapy [5]. In a randomised trial involving 257 adult AD patients, proactive therapy using tacrolimus significantly reduced disease exacerbations compared with vehicle treatment [9]. However, treatment was not successful in all patients, as approximately 20% of those treated with tacrolimus experienced disease exacerbations within 2 weeks of beginning treatment, and 30.2% of patients discontinued its use. Therefore, the characteristics of AD patients who benefit from TCI proactive therapy need to be identified.

In this study, we stratified AD patients who are more likely or less likely to respond to TCI proactive therapy and explored patient features that predict its success or failure. Patients were initially enrolled in the single-arm open-label clinical study to evaluate the efficacy of TCI proactive therapy. Thereafter, we analysed the correlation between those treatment outcomes and the patients’ clinical and laboratory findings.

## Materials and methods

### Prospective clinical study to evaluate the efficacy of TCI proactive therapy

A single-arm, open-label clinical study was conducted to evaluate the efficacy of proactive tacrolimus use in preventing skin rash exacerbation in AD patients who had already undergone remission-induction therapy with TCS for active eczematous lesions between April 2013 and January 2015. The study consisted of screening, remission-induction therapy, maintenance therapy, and proactive therapy periods. Patients were evaluated every 4 weeks, and their disease activity was assessed each time by two dermatologists (H.K. and T.E.) using the SCORing Atopic Dermatitis (SCORAD; score range 0–103) index [10]. Primary outcome of this prospective study was the efficacy of the proactive treatment, number of disease exacerbations (0 or 1), and time to the first disease exacerbation. The protocol was approved by Keio University School of Medicine Ethics Committee (Approval Number 20120124), conducted according to all relevant requirements from the Declaration of Helsinki, and registered in the UMIN Clinical Trial Registry (UMIN000017935). Written informed consent was obtained from all enrolled patients.

The participants were screened from a population of follow-up patients receiving standard AD treatment of emollient and TCS application. The inclusion criteria were the age of at least 18 years, AD diagnosis according to diagnostic criteria of Hanifin and Rajka [11] and history of reactive treatment (i.e. intermittent application of anti-inflammatory drugs) with TCS for more than 6 months at Keio University Hospital (Tokyo, Japan). Patients were excluded if they were pregnant or trying to conceive, had received systemic corticosteroids, immunosuppressants, or had ultraviolet light treatment 4 weeks prior to enrolment, were sensitive to 0.1% tacrolimus ointment (TACo), or had a severe coexisting disease or history of malignancy.

**Figure 1.**
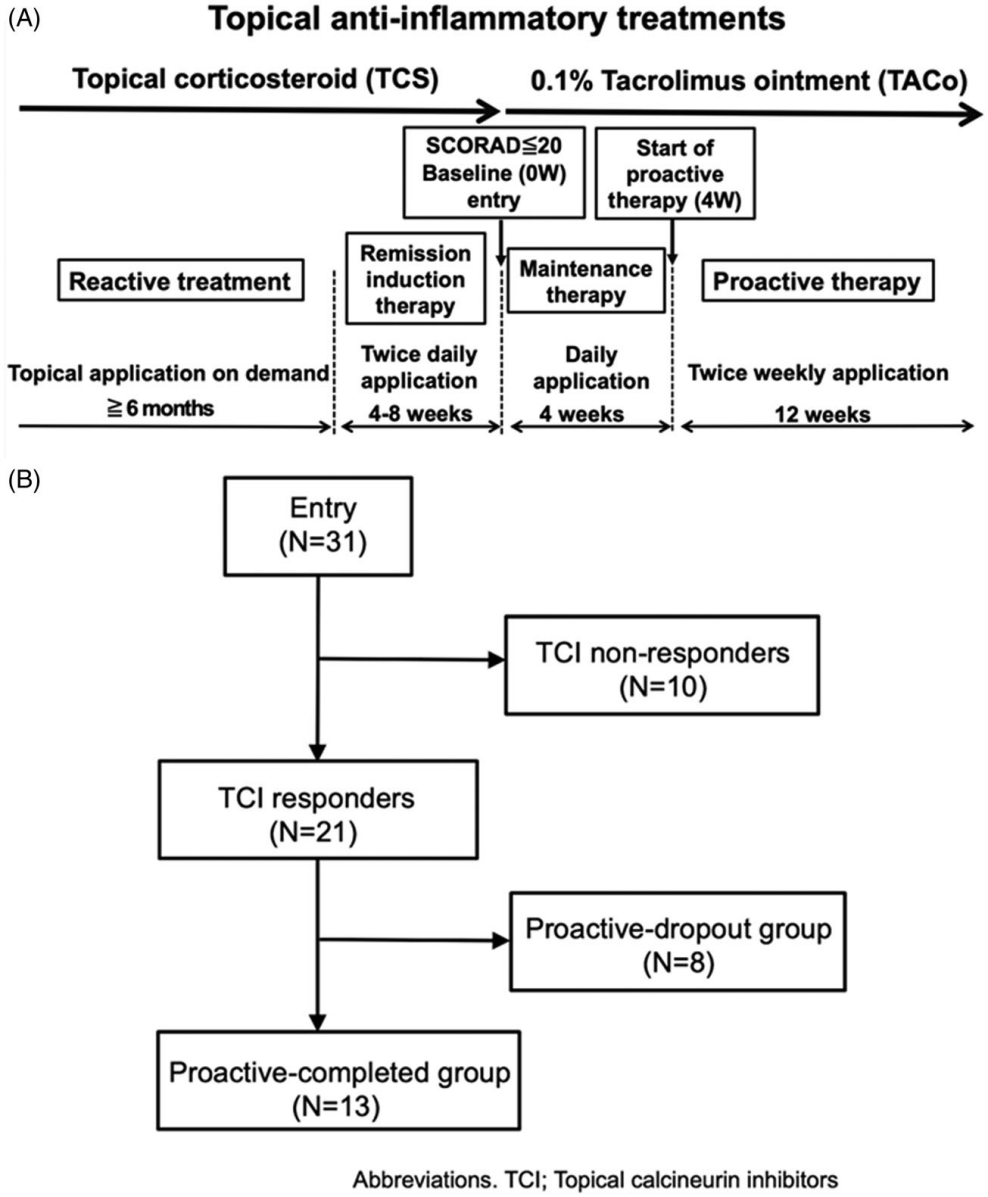
The study protocol and patient flow diagram. (A) Study protocol. The study consisted of screening, remission-induction therapy, maintenance therapy, and proactive therapy periods. During the screening period, patients were followed-up with standard reactive treatment (emollient and TCS application). During the remission-induction therapy period, patients applied medium-high to high-potency TCS twice daily for 4–8 weeks to the affected area. The baseline was defined as the first visit more than 4 weeks after the initiation of induction therapy, and patients with a SCORAD score of less than 20 moved to the maintenance period, during which they applied TACo once daily for 4 weeks to the previously affected area. Patients who completed the maintenance therapy period without lesion exacerbation or severe adverse events were moved to the proactive therapy period, during which they applied TACo twice weekly to the previously affected area for 12 weeks. During the maintenance and proactive therapy periods, lesion exacerbation was defined as an increase in the SCORAD score of 1.5-fold compared with baseline. Patients dropped out if they needed to apply TCS for more than 14 days to flare-up lesions or if they discontinued TACo application by their own decision because the lesion was worsening. The exacerbated lesions were treated with TCS twice daily until remission was achieved. (B) Patient flow diagram. All 31 patients enrolled in the study completed the remission-induction period. Among them, 21 completed the maintenance therapy (TCI responders), while the remaining 10 dropped out (TCI non-responders). Of the 21 TCI responders, 13 completed the proactive therapy (proactive-completed group) and the remaining 8 dropped out (proactive-dropout group). Of the patients in the TCI non-responders, 6 (19.4%) exhibited a SCORAD score increase of more than 1.5-fold above baseline at the end of treatment, 2 (6.5%) withdrew at their discretion, and 2 (6.5%) used TCS for more than 14 days by the end of the maintenance period. In the proactive-dropout group, 6 (19.4%) patients used TCS for more than 14 days, and 2 (6.5%) exhibited a SCORAD score increase of more than 1.5-fold above baseline by the end of treatment.

During the remission-induction therapy period, patients applied medium-high to high-potency TCSs to active skin lesions twice daily (b.i.d.) for 4–8 weeks. The baseline was defined as the first visit more than 4 weeks after the initiation of induction therapy, and patients with a SCORAD score of less than 20 received treatment of the subsequent study protocol. In the maintenance therapy period, patients applied TACo once daily for 4 weeks to the affected skin area and patients who completed the maintenance therapy period without lesion exacerbation or severe adverse events (AEs) began the proactive therapy period. During this period, patients applied TACo twice weekly to the affected area for 12 weeks (Figure 1(A)). During the maintenance and proactive therapy periods, patients could use TCS b.i.d. up to 14 days for any transient flare-ups of skin symptoms to maintain remission. Maintenance and/or proactive therapy was discontinued if patients met any following exacerbation criterion: 1.5-fold increase in the SCORAD score compared with baseline, a requirement for TCS application for more than 14 days for flare-up lesions, and the decision of patients themselves to discontinue TACo application because the lesion worsened. Oral administration of antihistamines was permitted over the entire treatment period, given that the type or dose was not changed.

### Laboratory data

Laboratory data analysed in this study were Thymus and activation-regulated chemokine (TARC), IgE, lactate dehydrogenase (LDH), and the peripheral eosinophil count. These were measured at each patient visit. Levels of TARC, IgE, and LDH were measured using the Allerport TARC reagent (Shionogi, Osaka, Japan), ImmunoCAP test (Thermo Fisher Diagnostics, Tokyo, Japan), and Iatro-LQ LDH rate-II(LSI Medience, Tokyo, Japan), respectively. The peripheral eosinophil count was performed using the XN-9000 Haematology Analyser (Sysmex, Hyogo, Japan).

### Statistical analyses

The results are expressed as the mean ± standard deviation. The SCORAD scores and biomarker levels (IgE, TARC, LDH, and peripheral eosinophil count) in each group were compared using Welch’s *t*-test. Adjustments for multiple comparisons were made using Holm’s method. In all analyses, *p* < 0.05 was considered statistically significant. The analyses were conducted in R (version 3.6.2) [12], and the figures were created using the ggplot2 package [13].

## Results

### Efficacy and safety of TCI proactive therapy in the prospective clinical study

Patient characteristics of the clinical study are summarised in Table 1 (detailed patient information is summarised in Supplemental Table 1). Of the 31 patients enrolled in this study, 21 (67.7%) completed the maintenance therapy (TCI responders) and the remaining 10 (32.3%) dropped out (TCI non-responders). Of the 21 TCI responders, 13 (41.9%) completed the proactive therapy (proactive-completed group) and the remaining 8 (25.8%) dropped out (proactive-dropout group) (Figure 1(B)). Meantime to disease exacerbation in TCI non-responders was 24.1 ± 5.1 days from the start of maintenance therapy, and that in the proactive-dropout group was 43.8 ± 16.8 days from the start of proactive therapy (Patient demographics of the proactive-completed and proactive-dropout groups are summarised in Table 2, and individual patient outcomes are summarised in Supplemental Table 2).

**Table 1.** Demographics of the patients at the baseline (*n* = 31).

Factor	All (*N* = 31)	TCI responders (*N* = 21)	TCI non-responders (*N* = 10)
Age (years)	35.1 ± 9.5	35.5 ± 9.6	34.3 ± 9.8
Sex (male), n (%)	21 (67.7)	13 (61.9)	8 (80)
SCORAD	13.1 ± 3.9	13.7 ± 3.7	12.0 ± 4.2
IgE (IU/mL)	4251.3 ± 4217.6 (*N* = 30^†^)	5126.5 ± 4718.3 (*N* = 20^†^)	2500.9 ± 2283.1
TARC (pg/mL)	1396.7 ± 1100.5	1469.7 ± 1193.9	1243.6 ± 911.5
LDH (U/L)	228.3 ± 45	233.7 ± 50.5	217.0 ± 29.6
Eosino (/μL)	394.4 ± 244.6	408.0 ± 272.7	365.8 ± 181

mean ± SD.

^†^Examination of IgE was not performed in 1 patient.

Abbreviations. SCORAD; SCORing Atopic Dermatitis, IgE; Immunoglobulin E, TARC; Thymus and activation-regulated chemokine, LDH; Lactate dehydrogenase, Eosino; Eosinophil, TCI; Topical calcineurin inhibitors.

**Table 2.** Demographics of the patients at the start of proactive therapy (*n* = 21).

Factor	TCI responders (*N* = 21)	Proactive-completed (*N* = 13)	Proactive-dropout (*N* = 8)
Age(years),	35.5 ± 9.6	36.5 ± 10.1	31.9 ± 7.9
Sex(male), n (%)	13 (61.9)	6 (46.2)	7 (87.5)
SCORAD	13.6 ± 5	11.7 ± 4.6	16.6 ± 4.2
IgE (IU/mL)	5309 ± 5396.3 (*N* = 20^†^)	3634.2 ± 2680.5 (*N* = 12^†^)	7821.3 ± 7465
TARC (pg/mL)	1829.8 ± 1821.2 (*N* = 20^†^)	902.5 ± 638.8 (*N* = 12^†^)	3220.8 ± 2162.5
LDH (U/L)	249.8 ± 48.2 (*N* = 20^†^)	232.3 ± 50.8 (*N* = 12^†^)	275.9 ± 31.3
Eosino(N) (/μL)	514.9 ± 312.6 (*N* = 20^†^)	356.8 ± 193.4 (*N* = 12^†^)	752.1 ± 315.1

mean ± SD.

^†^Examination was not performed in 1 patient.

Abbreviations. SCORAD; SCORing Atopic Dermatitis, IgE; Immunoglobulin E, TARC; Thymus and activation-regulated chemokine, LDH; Lactate dehydrogenase, Eosino; Eosinophil, TCI; Topical calcineurin inhibitors.

**Figure 2.**
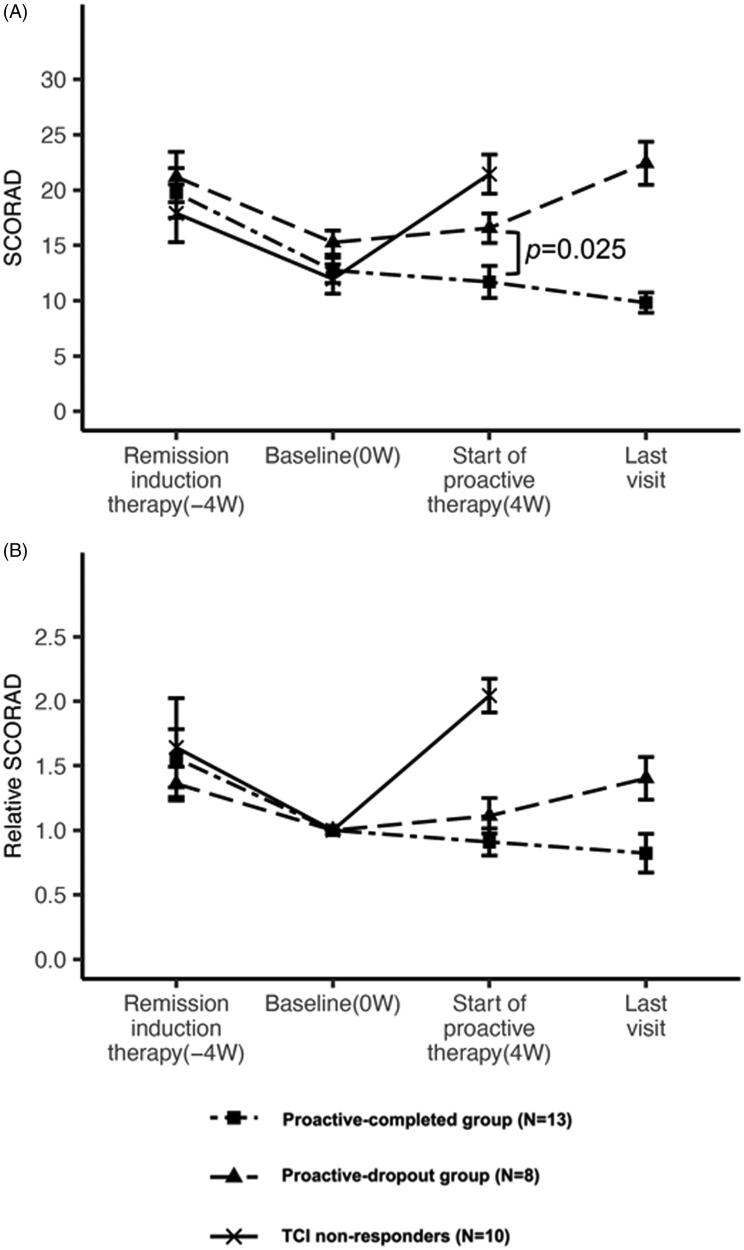
Change in the mean SCORAD and mean relative SCORAD scores during the treatment. (A) Changes in the mean SCORAD score in each treatment group. The mean SCORAD scores at the beginning of the remission-induction period and baseline did not differ significantly among the three treatment response groups. At the beginning of the remission-induction period, the mean SCORAD score was 17.9 ± 8.3 in the TCI non-responders, 19.7 ± 7.1 in the proactive-completed group, and 21.2 ± 7.2 in the proactive-dropout group. At baseline, the mean SCORAD score was 12.0 ± 4.2 in the TCI non-responders, 12.7 ± 3.6 in the proactive-completed group, and 15.3 ± 3.4 in the proactive-dropout group (no significant difference: adjusted *p* > 0.05 in all pairwise comparisons). At the end of maintenance therapy, the mean SCORAD score was 21.4 ± 5.5 in TCI non-responders, 11.7 ± 4.6 in the proactive-completed group, and 16.6 ± 4.2 in the proactive-dropout group (significantly different between the proactive-completed and proactive-dropout group; *p* = 0.025). At the last visit for proactive therapy, the mean SCORAD score was 9.8 ± 2.9 in the proactive-completed group and 22.4 ± 6.1 in the proactive-dropout group. (B) Changes in the mean relative SCORAD score in each treatment response group. The ratio of the SCORAD score at each visit relative to the baseline SCORAD score was calculated for each patient, and the changes in their mean values are shown. At the beginning of the remission-induction period, the mean relative SCORAD score was 1.64 in the TCI non-responders, 1.56 in the proactive-completed group, and 1.36 in the proactive-dropout group. At the end of maintenance therapy, the mean relative SCORAD score was 2.04 in the TACo-dropout group, 0.91 in the proactive-completed group, and 1.11 in the proactive-dropout group. At the last visit for proactive therapy, the mean SCORAD score was 0.82 in the proactive-completed group and 1.4 in the proactive-dropout group.

The mean improvement rate in the SCORAD score at baseline compared with the beginning of the remission-induction period was 30.8% in the proactive-completed group, 24.1% in the proactive-dropout group, and 12.1% in the TCI non-responders (no significant difference: adjusted *p* > 0.05 in all pairwise comparisons). The mean SCORAD scores at baseline in the proactive-completed, proactive-dropout, and TCI non-responders were 12.7 ± 3.6, 15.3 ± 3.4, and 12 ± 4.2, respectively. Although there was no significant difference among the three groups (adjusted *p* > 0.05 in all pairwise comparisons), the proactive-dropout group had a relatively higher mean SCORAD score (Figure 2).

The mean change in the SCORAD score at the end of maintenance therapy compared with baseline was a 2.0-fold increase in the TCI non-responders, 0.9-fold decrease in the proactive-completed group, and 1.1-fold increase in the proactive-dropout group. The mean SCORAD score at the start of proactive therapy was significantly different between the proactive-completed (11.7 ± 4.6) and proactive-dropout (16.6 ± 4.2) groups (*p* = 0.025). During the proactive therapy, the mean SCORAD score in the proactive-completed group decreased further to 9.8 ± 2.9 but increased in the proactive-dropout group to 22.4 ± 6.1 (Figure 2, Supplemental Table 3).

In the overall study population, AEs were observed in 7 patients (22.6%): 6 (19.4%) during maintenance therapy and 1 (3.2%) during proactive therapy. The observed AEs were TACo irritation (*n* = 3, 9.7%), pruritis (*n* = 3, 9.7%), folliculitis (*n* = 2, 6.5%), herpes simplex (*n* = 1, 3.2%), and hand swelling (*n* = 1, 3.2%). No serious AEs or treatment-related deaths occurred, and all AEs recovered.

### Laboratory findings predicting the efficacy of 0.1% tacrolimus ointment proactive use

To identify laboratory findings that predict the efficacy of TACo in preventing skin lesion exacerbations, serum levels of IgE, LDH, TARC, and peripheral eosinophil count were analysed at the beginning of remission induction therapy, baseline, and the beginning of proactive therapy. These data were subsequently correlated with treatment outcomes.

**Figure 3.**
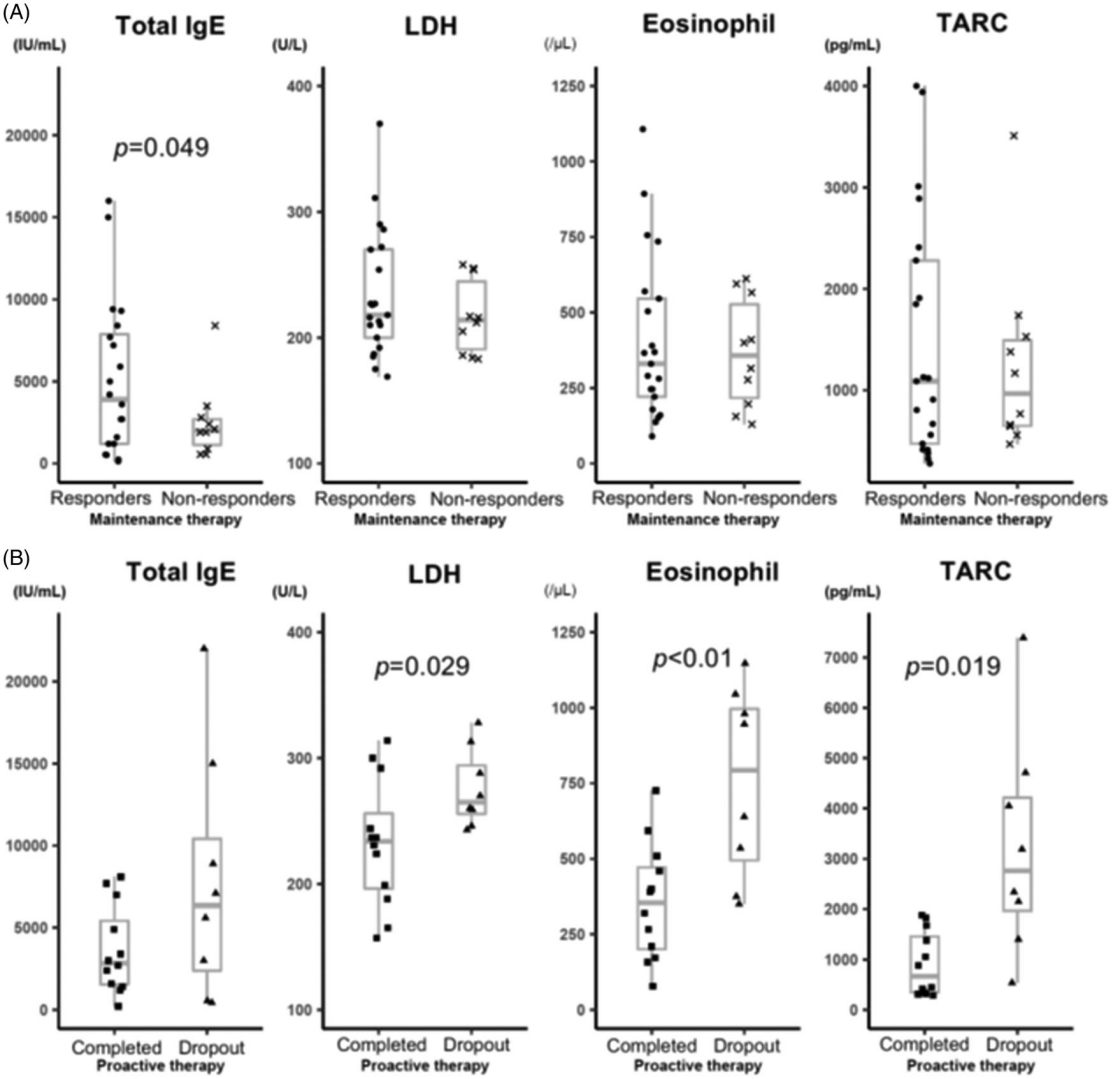
Laboratory marker levels according to the treatment outcome. Serum IgE, LDH and TARC levels, and peripheral eosinophil count were analysed at baseline and the beginning of the proactive therapy period. The correlations between these levels and the clinical outcomes of AD patients after TACo maintenance and proactive therapy were evaluated. (A) Laboratory marker levels between the TCI responders and TCI non-responders at baseline. The mean serum IgE level was significantly higher (*p* = 0.049) in the TCI responders (5126.5 ± 4718.3 IU/mL) than in non-responders (2500.9 ± 2283.1 IU/mL). In contrast, the other biomarkers were not significantly different. The mean LDH level at baseline was 233.7 ± 50.5 U/L in the TCI responders and 217 ± 29.6 U/L in the TCI non-responders (*p* = 0.257). The mean TARC level was 1469.7 ± 1193.9 pg/ml in the TCI responders and 1243.6 ± 911.5 pg/ml in the TCI non-responders (*p* = 0.566). The mean eosinophil count was 408 ± 272.7/μL in the TCI responders and 365.8 ± 181/μL in the TCI non-responders(*p* = 0.614). (B) Laboratory marker levels between the proactive-completed and proactive-dropout groups at the start of the proactive therapy period. Mean LDH and TARC levels and eosinophil count significantly differed between the proactive-completed and proactive-dropout groups. More specifically, the mean LDH level at the start of proactive therapy was 232.3 ± 50.8 U/L in the proactive-completed group and 275.9 ± 31.3 U/L in the proactive-dropout group (*p* = 0.029). The mean TARC level was 902.5 ± 638.8 pg/ml in the proactive-completed group and 3220.8 ± 2162.5 pg/ml in the proactive-dropout group (*p* = 0.019). The mean eosinophil count was significantly lower (*p* < 0.01) in the proactive-completed group (356.8 ± 193.4/μL) than in the proactive-dropout group (752.1 ± 315.1/μL). In contrast, the mean serum IgE level did not differ significantly (*p* = 0.165) between the proactive-completed group (3634.2 ± 2680.5 IU/mL) and the proactive-dropout group (7821.3 ± 7465 IU/mL).

In the analysis at baseline, mean IgE levels were significantly different (*p* = 0.049) between TCI responders (5126.5 ± 4718.3 IU/mL; *n* = 21) and TCI non-responders, (2500.9 ± 2283.1 IU/mL; *n* = 10). In contrast, the other laboratory findings did not significantly differ between the two groups (Figure 3(A), Supplemental Table 3). In the analysis between proactive-completed (*n* = 12; one patient was not assayed at this visit) and the proactive-dropout (*n* = 8) groups, LDH (*p* = 0.029), TARC levels (*p* = 0.019), and eosinophil count (*p* < 0.01) significantly differed at the start of proactive therapy. In contrast, the mean IgE levels were comparable between both groups (Figure 3(B), Supplemental Table 5).

**Figure 4.**
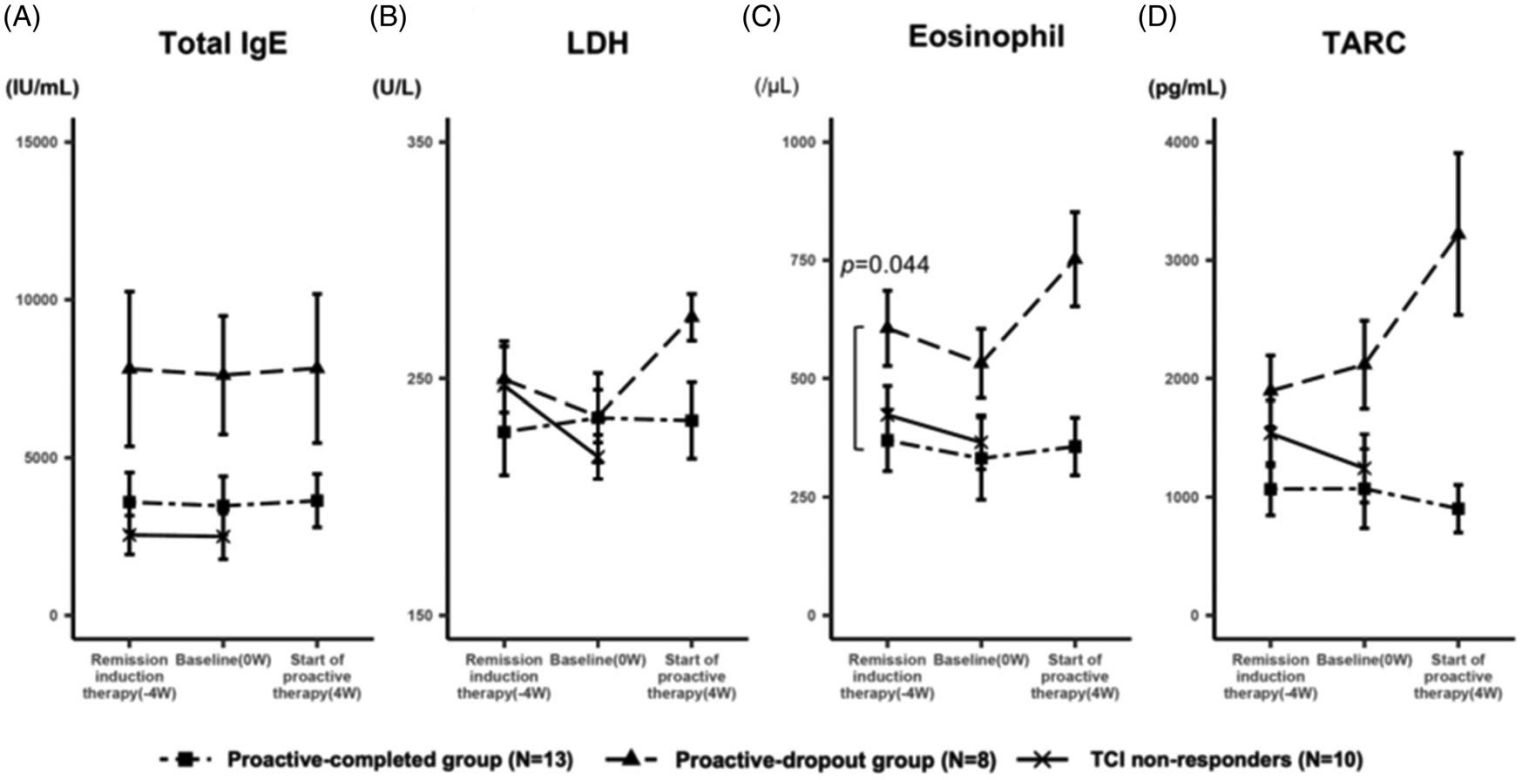
Change in the level of laboratory markers during the treatment. (A) The transition of the mean serum IgE levels. At the start of remission induction therapy, the mean IgE levels were 3588.3 ± 2962 IU/mL, 7801.4 ± 7736.7 IU/mL, and 5140.5 ± 5447.3 IU/mL in the proactive-completed, proactive-dropout, and TCI non-responders, respectively (*p* = 0.21; between proactive-completed and proactive-dropout groups). The mean IgE levels of each group were similar throughout the study, regardless of the treatment regimen or response. (B) The transition of the mean serum LDH levels. At the start of remission induction therapy, the mean LDH levels were 227.5 ± 58.3 U/L and 249.6 ± 44 U/L in the proactive-completed and proactive-dropout groups, respectively (*p* = 0.34). During the remission induction therapy period, LDH levels decreased in the proactive-dropout group and slightly increased in the proactive-completed group. During the maintenance therapy period, LDH levels increased markedly in the proactive-dropout group while slightly decreasing in the proactive-completed group. (C) The transition of the mean eosinophil count in peripheral blood. At the start of remission induction therapy, the mean eosinophil count was 369.8 ± 204.9/μL and 606.3 ± 251.9/μL in the proactive-completed and proactive-dropout groups, respectively (*p* = 0.044). During the remission induction therapy period, eosinophil count decreased in both proactive-dropout and proactive-completed groups. During the maintenance therapy period, eosinophil count markedly increased in the proactive-dropout group and slightly increased in the proactive-completed group. (D) The transition of the mean serum TARC levels. At the start of remission induction therapy, the mean TARC levels were 1068.2 ± 701 pg/mL and 1894.8 ± 947.7 pg/mL in the proactive-completed and proactive-dropout groups, respectively (*p* = 0.056) . During the remission induction therapy period, TARC levels increased in the proactive-dropout group and about the same in the proactive-completed group. During the maintenance therapy period, TARC levels further increased in the proactive-dropout group while decreased in the proactive-completed group.

Next, we examined the transition of these laboratory markers in the proactive-completed and proactive-dropout groups. The mean IgE levels of each group were similar throughout the study, regardless of treatment regimen or response, and that of the proactive-completed group tended to be lower (Figure 4(A)). The mean LDH levels were comparable between the proactive-completed and proactive-dropout groups at the baseline. However, the proactive-dropout group showed an increase during the maintenance therapy period (Figure 4(B)). The mean eosinophil count was significantly lower in the proactive-completed group (369.8 ± 204.9/μL; *n* = 13) compared to proactive-dropout (606.3 ± 251.9/μL; *n* = 8) group at the start of remission induction therapy (*p* < 0.05). During the remission induction therapy period, it decreased in both groups. However, the proactive-dropout group showed an increase during the maintenance therapy period (Figure 4(C)). The mean TARC level tended to be lower in the proactive-completed group at the start of remission induction therapy. In the proactive-dropout group, it increased during the remission induction therapy period and further increased during the maintenance therapy period, while it was about the same or decreased in the proactive-completed group (Figure 4(D)).

In the further analysis of TCI responders, the success rate of TCI-proactive therapy in patients with a TARC level ≤ 2000 pg/mL at the start of the proactive therapy was 85.7% (12/14 patients), while no patients (0/6 patients) with a TARC level >2000 pg/mL succeeded (Supplemental Figure 2A). The success rate of patients whose TARC levels decreased during maintenance therapy was 75% (6/8 patients), while patients whose TARC levels increased was 50% (6/12 patients) (Supplemental Figure 2B). In particular, all patients (4/4) with TARC levels that increased by more than 1000 pg/mL failed the proactive therapy.

## Discussion

AD shows highly variable clinical manifestations, biomarkers, and treatment responses depending on the patient’s age, genetic background, environmental stimuli, and disease activity [2]. In particular, treatment responses may differ among various therapies, even in patients with similar clinical presentations. For instance, 28.3% of patients treated with TCI reactive therapy failed to show a 50% or better improvement [14]. In a clinical trial of the topical application of crisaborole, a phosphodiesterase-4 inhibitor, almost half of the patients failed to achieve a ‘clear’/‘almost clear’ score on the Investigator’s Static Global Assessment scale [15]. These findings suggest that not all AD patients benefit from the same treatments; therefore, treatments should be individualised, and indicators that can predict the efficacy of each therapy should be identified.

In this study, we first stratified AD patients by the response types to TCI proactive therapy in a prospective clinical study, which showed three different types: TCI non-responders, proactive-dropout, and proactive-completed groups. At the baseline, the proactive-dropout group had a relatively higher mean SCORAD score. The other two groups showed similar SCORAD scores at baseline, but the TCI non-responders showed a rapid worsening of symptoms after entering the maintenance phase, while the proactive-completed group showed a further decrease in SCORAD scores. These results suggest that the TCI non-responders include patients for whom TCI itself was ineffective, the proactive-dropout group include patients for whom TCI is effective but disease activity was inadequately controlled during remission-induction therapy with TCS and its relapses with prolongation of TCI dosing interval, and the proactive-completed group includes patients for whom TCI is effective and disease control is possible with prolonged dosing intervals.

Next, we correlated these outcomes with patients’ clinical and laboratory features to explore findings that predict the efficacy of TCI proactive therapy. As a result, a lower IgE level at baseline significantly correlated with the failure of daily TACo application. While 80% of AD patients have an increase in the serum total IgE level, that in the remaining 20% is normal [1]. These latter patients are considered to have intrinsic AD, which usually has a relatively late onset, a higher susceptibility in females, and milder severity. Immunologically, it is characterised by the absence of specific IgE, lower expression levels of IL-4, IL-5, and IL-13, and higher expression of interferon-γ [16]. On the other hand, the principal pharmacological action of tacrolimus in AD is to suppress T-cell activation by inhibiting IL-4 and IL-5 production, which in turn reduces IgE production in B cells and mast cells degranulation. Altogether, the results from those studies and the present work indicate that TCI is useful for preventing skin rash exacerbations in AD patients with intimate involvement of IgE in their pathogenesis.

This study also showed significant differences in the mean SCORAD score, peripheral eosinophil count, serum LDH and TARC levels between the proactive-completed and proactive-dropout groups at the start of proactive therapy. Those were significantly higher in a proactive-dropout group at the beginning of proactive therapy, and further elevated during the maintenance therapy period. SCORAD is the most widely used evaluation tool by clinicians as an indicator of disease activity in AD, and its value correlates with the disease severity [17]. The number of peripheral eosinophils, which are produced mainly by IL-5 stimulation, tends to increase with disease severity [3]. The serum LDH level reflects tissue damage caused by skin inflammation, which is associated with disease severity [3]. The serum TARC level is highly correlated with the Eczema Area and Severity Index score, which is the second most widely used tool to evaluate disease activity following SCORAD, and is now regarded as the most sensitive clinical biomarker of AD [17,18]. These findings support that the reason for treatment failure in the proactive-dropout group was inadequate remission induction with TCS, resulting in residual inflammation of an intensity that could not be controlled by the potency of tacrolimus during the maintenance therapy period.

Although the SCORAD assessment is a good indicator for measuring disease activity in clinical practice, its value may vary among evaluators because it is a subjective scale. Therefore, combining SCROAD and laboratory findings can provide a more accurate picture of disease activity. Among the laboratory markers we analysed, the elevation of TARC levels had best reflected the treatment failure. In the proactive-dropout group, the mean TARC, LDH, and eosinophil were all elevated during the maintenance therapy period, indicating that those are useful in assessing lesion exacerbation. On the other hand, only the mean TARC level showed an increasing trend in the remission induction period of the proactive-dropout group, suggesting that some of the patients in this group had subclinical inflammation that had not been adequately controlled by TCS. Together with the findings that the success rate of TCI proactive therapy was lower in patients whose TARC level increased during the maintenance therapy, TARC will be the most sensitive laboratory marker to evaluate the intensity of subclinical inflammation.

This study had several limitations. First, this clinical study was conducted as a single-arm open-label design, hence we cannot exclude physician subjectivity in those outcomes. Second, deviations from the true outcome values cannot be ruled out as the number of patients enrolled in this study was small. Third, exploration of clinical and laboratory findings that predict the outcome of treatment is based on the results of a clinical study and those significances are not verified. Finally, this study included only Japanese AD patients, and ethnic variations should be considered when interpreting the results.

In conclusion, this study suggested that patients with low IgE may have a less response to TCI, and adequate control of disease activity is an important factor for the success of TCI proactive therapy. SCORAD is a useful scale in assessing disease activity, and when combined with laboratory findings such as peripheral eosinophil count, serum LDH and TARC levels, it can provide a more accurate assessment of disease activity and help to determine whether TCI proactive therapy is indicated.

## Supplementary Material

Supplemental Material

## Data Availability

The authors confirm that the data supporting the findings of this study are available within the article [and/or] its Supplementary materials.

## Abbreviations

AD: Atopic dermatitis
FDA: The U.S. Food and Drug Administration
IgE: Immunoglobulin E
LDH: Lactate dehydrogenase
TACo: 0.1% tacrolimus ointment
TARC: Thymus and activation-regulated chemokine
TCI: Topical calcineurin inhibitors
TCS: Topical corticosteroids
SCORAD: SCORing Atopic Dermatitis.

## References

[1] Eichenfield LF, Tom WL, Chamlin SL, et al. Guidelines of care for the management of atopic dermatitis: section 1. Diagnosis and assessment of atopic dermatitis work group. J Am Acad Dermatol. 2014;70(2):338–351. 24290431 10.1016/j.jaad.2013.10.010 PMC4410183

[2] Langan S, Irvine A, Weidinger S. Atopic dermatitis. Lancet. 2020;396(10247):345–360. 32738956 10.1016/S0140-6736(20)31286-1

[3] Katoh N, Ohya Y, Ikeda M, et al. Clinical practice guidelines for the management of atopic dermatitis 2018. J Dermatol. 2019;46(12):1053–1101. 31599013 10.1111/1346-8138.15090

[4] Eichenfield LF, Tom WL, Berger TG, et al. Guidelines of care for the management of atopic dermatitis: section 2. Management and treatment of atopic dermatitis with topical therapies. J Am Acad Dermatol. 2014;71(1):116–132. 24813302 10.1016/j.jaad.2014.03.023 PMC4326095

[5] Wollenberg A, Barbarot S, Bieber T, et al. Consensus-based european guidelines for treatment of atopic eczema (atopic dermatitis) in adults and children: part I. J Eur Acad Dermatol Venereol. 2018;32(5):657–682. 29676534 10.1111/jdv.14891

[6] Hoare C, Li Wan Po A, Williams H. Systematic review of treatments for atopic eczema. Health Technology Assessment. 2000;4(37):1–191. PMC4782813 11134919

[7] Nakahara T, Morimoto H, Murakami N, et al. Mechanistic insights into topical tacrolimus for the treatment of atopic dermatitis. Pediatr Allergy Immunol. 2018;29(3):233–238. 29205511 10.1111/pai.12842

[8] Czarnecka-Operacz M, Jenerowicz D. Topical calcineurin inhibitors in the treatment of atopic dermatitis – an update on safety issues. J Dtsch Dermatol Ges. 2012;10(3):167–172. 21974750 10.1111/j.1610-0387.2011.07791.x

[9] Wollenberg A, Reitamo S, Girolomoni G, et al. Proactive treatment of atopic dermatitis in adults with 0.1% tacrolimus ointment. Allergy. 2008;63(7):742–750. 18592619

[10] European Task Force on Atopic Dermatitis. Severity scoring of atopic dermatitis: the scorad index. Dermatology. 1993;186(1):763–769. 10.1159/000247298 8435513

[11] Hanifin J, Rajka D. Diagnostic features of atopic dermatitis. Acta Dermato-Venereologica. 1980;92(Suppl):44–47.

[12] R Core Team 2019. “R: A Language and Environment for Statistical Computing.” Vienna, Austria: R Foundation for Statistical Computing. Available from: https://www.r-project.org/

[13] Wickham H. 2016. “ggplot2: Elegant Graphics for Data Analysis.” Springer-Verlag New York. Available from: https://ggplot2.tidyverse.org/

[14] Hanifin JM, Ling MR, Langley R, et al. Tacrolimus ointment for the treatment of atopic dermatitis in adult patients: part I, efficacy. Journal of the American Academy of Dermatology. 2001;44(1):S28–S38. 11145793 10.1067/mjd.2001.109810

[15] Paller AS, Tom WL, Lebwohl MG, et al. Efficacy and safety of crisaborole ointment, a novel, nonsteroidal phosphodiesterase 4 (PDE4) inhibitor for the topical treatment of atopic dermatitis (AD) in children and adults. J Am Acad Dermatol. 2016;75(3):494–503.e6. 27417017 10.1016/j.jaad.2016.05.046

[16] Tokura Y. Extrinsic and intrinsic types of atopic dermatitis. J Dermatol Sci. 2010;58(1):1–7. 20207111 10.1016/j.jdermsci.2010.02.008

[17] Iannone M, Tonini G, Janowska A, et al. Definition of treatment goals in terms of clinician-reported disease severity and patient-reported outcomes in moderate-to-severe adult atopic dermatitis: a systematic review. Curr Med Res Opin. 2021;37(8):1295–1301. 34027753 10.1080/03007995.2021.1933929

[18] Kataoka Y. Thymus and activation-regulated chemokine as a clinical biomarker in atopic dermatitis. J Dermatol. 2014;41(3):221–229. 24628072 10.1111/1346-8138.12440

