# Supplementary material for: Stratification of atopic dermatitis patients by patterns of response to proactive therapy with topical tacrolimus: low serum IgE levels and inadequately controlled disease activity at the start of treatment predict its failure
Source: Ann Med. 2021 Nov 19;53(1):2207–16. doi: 10.1080/07853890.2021.2004319 (PMC8805968; doi:10.1080/07853890.2021.2004319)
Supplement: Supplemental Material [file IANN_A_2004319_SM9574.zip › Supplemental files/Supple Table1 .docx]

**Supplemental table1 Individual patient characteristics enrolled in the study.**

| Pt No. | Age | Sex | SCORAD | | | Baseline laboratory data | | | |
| --- | --- | --- | --- | --- | --- | --- | --- | --- | --- |
|  |  |  | Maximum score at the screening period | Before induction therapy | Baseline | IgE  (IU/mL) | LDH  (U/L) | Eosino  (/μL) | TARC  (pg/mL) |
| 1 | 41 | M | 35 | 35 | 16.5 | 2400 | 183 | 197 | 769 |
| 2 | 31 | F | 21.5 | 21.5 | 10 | 130 | 216 | 369 | 669 |
| 3 | 26 | F | 28.5 | 28.5 | 18 | NA | 185 | 246 | 279 |
| 4 | 48 | M | 25.5 | 25.5 | 20 | 15000 | 254 | 366 | 1850 |
| 5 | 38 | F | 35.5 | 21 | 17.5 | 2700 | 175 | 221 | 326 |
| 6 | 23 | M | 28.5 | 12 | 12 | 8400 | 186 | 410 | 662 |
| 7 | 47 | F | 32 | 25.5 | 13 | 5000 | 192 | 152 | 379 |
| 8 | 21 | M | 31 | 31 | 13 | 8400 | 286 | 179 | 909 |
| 9 | 31 | M | 21 | 16.5 | 11 | 3600 | 370 | 546 | 3940 |
| 10 | 51 | M | 17.5 | 10 | 10 | 240 | 272 | 90 | 413 |
| 11 | 26 | M | 29.5 | 29.5 | 17 | 540 | 227 | 390 | 1090 |
| 12 | 37 | M | 38 | 23 | 16.5 | 520 | 226 | 893 | 561 |
| 13 | 41 | M | 25.5 | 21.5 | 16.1 | 2700 | 187 | 160 | 806 |
| 14 | 41 | M | 29 | 26 | 18 | 7700 | 290 | 290 | 1120 |
| 15 | 24 | M | 27 | 23.5 | 17 | 16000 | 311 | 330 | 4000 |
| 16 | 43 | M | 31.5 | 18.6 | 8.1 | 3500 | 258 | 566 | 3510 |
| 17 | 25 | M | 24.5 | 21.5 | 9 | 2800 | 205 | 277 | 1170 |
| 18 | 31 | M | 15.5 | 11 | 7.5 | 1600 | 169 | 137 | 474 |
| 19 | 30 | F | 14.5 | 9 | 9 | 7200 | 213 | 1107 | 2280 |
| 20 | 43 | M | 28.5 | 28.5 | 16.5 | 9400 | 210 | 281 | 1130 |
| 21 | 33 | M | 23 | 17.5 | 17.5 | 2100 | 254 | 130 | 646 |
| 22 | 22 | M | 21 | 17 | 14.5 | 4200 | 218 | 756 | 2890 |
| 23 | 20 | M | 26.5 | 13 | 9.4 | 890 | 255 | 595 | 1740 |
| 24 | 45 | F | 12.5 | 12.5 | 9 | 560 | 212 | 400 | 1530 |
| 25 | 38 | M | 14.5 | 10 | 9 | 1900 | 216 | 612 | 1380 |
| 26 | 55 | F | 20.6 | 20.6 | 10 | 1200 | 270 | 246 | 417 |
| 27 | 34 | M | 20 | 11 | 11 | 9300 | 200 | 504 | 3010 |
| 28 | 28 | F | 24.4 | 24.4 | 9.6 | 1900 | 184 | 156 | 470 |
| 29 | 37 | F | 14 | 11.5 | 9.6 | 5900 | 227 | 735 | 2410 |
| 30 | 47 | M | 27 | 6.5 | 19.5 | 559 | 217 | 315 | 559 |
| 31 | 31 | F | 17.5 | 14.5 | 12.5 | 1200 | 210 | 570 | 1910 |

Abbreviations. SCORAD; SCORing Atopic Dermatitis, IgE; Immunoglobulin E, LDH; Lactate dehydrogenase,

Eosino; Eosinophil, TARC; Thymus and activation-regulated chemokine, NA; Not available
