# Supplementary material for: Stratification of atopic dermatitis patients by patterns of response to proactive therapy with topical tacrolimus: low serum IgE levels and inadequately controlled disease activity at the start of treatment predict its failure
Source: Ann Med. 2021 Nov 19;53(1):2207–16. doi: 10.1080/07853890.2021.2004319 (PMC8805968; doi:10.1080/07853890.2021.2004319)
Supplement: Supplemental Material [file IANN_A_2004319_SM9574.zip › Supplemental files/Supple Table2 revised.docx]

**Supplemental Table 2: Individual patient outcomes in the single-arm open label clinical study**

| Pt No. | Time to disease exacerbation (days) | | Total amount of TACo application (g) | | Total amount of TCS application (g) | |
| --- | --- | --- | --- | --- | --- | --- |
|  | Maintenance period | Proactive period | Maintenance period | Proactive period | Maintenance period | Proactive period |
| TCI non-responders | | | | | | |
| 1 | 28 | − | 72 | − | 16 | − |
| 6 | 21 | − | 33.3 | − | 15 | − |
| 16 | 28 | − | 210 | − | 30 | − |
| 17 | 28 | − | 60 | − | 0 | − |
| 21 | 21 | − | 26.7 | − | 10 | − |
| 23 | 28 | − | 32 | − | 0 | − |
| 24 | 28 | − | 16 | − | NA | − |
| 25 | 14 | − | 20 | − | NA | − |
| 28 | 21 | − | 13.3 | − | 5 | − |
| 30 | 17 | − | NA | − | NA | − |
| Proactive-dropout group | | | | | | |
| 4 | − | 61 | 33.3 | 155 | 20 | 15 |
| 11 | − | 28 | 95 | 40 | 0 | 20 |
| 12 | − | 43 | 40 | 60 | 0 | 17.5 |
| 15 | − | 62 | 36.7 | 65 | 0 | 20 |
| 20 | − | 54 | 95 | 75 | 0 | 55 |
| 22 | − | 28 | 16.7 | 57.5 | 0 | 22.5 |
| 27 | − | 55 | 75 | 90 | 0 | 18 |
| 29 | − | 19 | 5 | - | 15 | − |
| Proactive-completed group | | | | | | |
| 2 | − | − | 33.3 | 50 | 3.3 | 10 |
| 3 | − | − | 24 | 52.5 | 0 | 0 |
| 5 | − | − | 42.5 | 42.5 | 0 | 0 |
| 7 | − | − | 4 | 12 | 0 | 0 |
| 8 | − | − | 20 | 35 | 0 | 11.2 |
| 9 | − | − | 85 | 110 | 0 | 3 |
| 10 | − | − | 40 | 42.5 | 0 | 0 |
| 13 | − | − | 53.6 | 70 | 4 | 7.5 |
| 14 | − | − | 17.1 | 70 | 1.1 | 0 |
| 18 | − | − | 40 | 30 | 0 | 5 |
| 19 | − | − | 32 | 95 | 0 | 0 |
| 26 | − | − | 32.5 | 30 | 0 | 0 |
| 31 | − | − | 40 | 50 | 0 | 75 |

Abbreviations. TCI; Topical calcineurin inhibitors, TACo; 0.1% tacrolimus ointment, TCS; Topical corticosteroids, NA; Not available
