# Supplementary material for: Stratification of atopic dermatitis patients by patterns of response to proactive therapy with topical tacrolimus: low serum IgE levels and inadequately controlled disease activity at the start of treatment predict its failure
Source: Ann Med. 2021 Nov 19;53(1):2207–16. doi: 10.1080/07853890.2021.2004319 (PMC8805968; doi:10.1080/07853890.2021.2004319)
Supplement: Supplemental Material [file IANN_A_2004319_SM9574.zip › Supplemental files/Supple Table3 revised.docx]

**Supplemental Table3**

**Comparison of** **biomarkers between the TCI responders and TCI non-responders at baseline**

| Factor | TCI responders  (N=21) | TCI non-responders  (N=10) | Difference of means | 95% CI^†^ | p value^†^ |
| --- | --- | --- | --- | --- | --- |
| SCORAD | 13.7±3.7 | 12±4.2 | 1.7 | -1.6 − 5.1 | 0.28 |
| IgE (IU/mL) | 5126.5±4718.3  (N=20^‡^) | 2500.9±2283.1 | 2625.6 | 6.9 − 5244.3 | 0.049* |
| TARC (pg/mL) | 1469.7±1193.9 | 1243.6±911.5 | 226.1 | -580 − 1030.1 | 0.57 |
| LDH (U/L) | 233.7±50.5 | 217±29.6 | 16.7 | -12.9 − 46.3 | 0.26 |
| Eosino(N) (/μL) | 408±272.7 | 365.8±181 | 42.2 | -127.1 − 212.1 | 0.61 |

^†^SCORAD and biomarkers of each group were compared using Welch’s t-test.

^‡^Examination was not performed in 1 patient.

**p*<0.05

Abbreviations. TCI; Topical calcineurin inhibitors, SCORAD; SCORing Atopic Dermatitis, IgE; Immunoglobulin E, TARC; Thymus and activation-regulated chemokine, LDH; Lactate dehydrogenase, Eosino; Eosinophil,
