# Supplementary material for: Stratification of atopic dermatitis patients by patterns of response to proactive therapy with topical tacrolimus: low serum IgE levels and inadequately controlled disease activity at the start of treatment predict its failure
Source: Ann Med. 2021 Nov 19;53(1):2207–16. doi: 10.1080/07853890.2021.2004319 (PMC8805968; doi:10.1080/07853890.2021.2004319)
Supplement: Supplemental Material [file IANN_A_2004319_SM9574.zip › Supplemental files/Supple Table4 .docx]

**Supplemental Table4**

**Comparison of biomarkers between the proactive-completed and proactive-dropout groups at the start of remission induction therapy**

| Factor | Proactive-completed  (N=13) | Proactive-  dropout (N=8) | Difference of means | 95% CI^†^ | p value^†^ |
| --- | --- | --- | --- | --- | --- |
| SCORAD | 19.7±7.1 | 21.2±7.2 | -1.4 | -8.3 − 5.4 | 0.66 |
| IgE (IU/mL) | 3588.3±2962  (N=12^‡^) | 7801.4±7736.7  (N=7^‡^) | -4213.1 | -11408.6 − 2982.4 | 0.21 |
| TARC (pg/mL) | 1068.2±701  (N=12^‡^) | 1894.8±947.7 | -826.6 | -1679.2 −26.1 | 0.056 |
| LDH (U/L) | 227.5±58.3 | 249.6±44 | -22.1 | -69.2 −25.1 | 0.34 |
| Eosino(N)(/μL) | 369.8±204.9 | 606.3±251.9 | -47 | -465.3 − 7.5 | 0.044* |

^†^ SCORAD and biomarkers of each group were compared using Welch’s t-test.

^‡^ Examination was not performed in 1 patient of each group.

**p*<0.05

Abbreviations. SCORAD; SCORing Atopic Dermatitis, IgE; Immunoglobulin E, TARC; Thymus and activation-regulated chemokine, LDH; Lactate dehydrogenase, Eosino; Eosinophil
