# Supplementary material for: Stratification of atopic dermatitis patients by patterns of response to proactive therapy with topical tacrolimus: low serum IgE levels and inadequately controlled disease activity at the start of treatment predict its failure
Source: Ann Med. 2021 Nov 19;53(1):2207–16. doi: 10.1080/07853890.2021.2004319 (PMC8805968; doi:10.1080/07853890.2021.2004319)
Supplement: Supplemental Material [file IANN_A_2004319_SM9574.zip › Supplemental files/Supple Table5 .docx]

**Supplemental Table5**

**Comparison of biomarkers between the proactive-completed and proactive-dropout groups at the start of proactive therapy**

| Factor | Proactive-completed  (N=13) | Proactive-  dropout (N=8) | Difference of means | 95% CI^†^ | p value^†^ |
| --- | --- | --- | --- | --- | --- |
| SCORAD | 11.7±4.6 | 16.6±4.2 | -4.9 | -9.01 − 0.7 | 0.025* |
| IgE (IU/mL) | 3634.2±2680.5  (N=12^‡^) | 7821.3±7465 | -4187.1 | -10500.5 − 2126.3 | 0.17 |
| TARC (pg/mL) | 902.5±638.8  (N=12^‡^) | 3220.8±2162.5 | -2318.3 | -4139.1 −  -497.4 | 0.019* |
| LDH (U/L) | 232.3±50.8 (N=12^‡^) | 275.9±31.3 | -43.6 | -82.1 −  -5.03 | 0.029* |
| Eosino(N)(/μL) | 356.8±193.4 (N=12^‡^) | 752.1±315.1 | -395.3 | -671.1 −  -119.6 | 0.009* |

^†^SCORAD and biomarkers of each group were compared using Welch’s t-test.

^‡^Examination was not performed in 1 patient.

**p*<0.05

Abbreviations. SCORAD; SCORing Atopic Dermatitis, IgE; Immunoglobulin E, TARC; Thymus and activation-regulated chemokine, LDH; Lactate dehydrogenase, Eosino; Eosinophil
