# Supplementary figures and images for: Stratification of atopic dermatitis patients by patterns of response to proactive therapy with topical tacrolimus: low serum IgE levels and inadequately controlled disease activity at the start of treatment predict its failure
Source: Ann Med. 2021 Nov 19;53(1):2207–16. doi: 10.1080/07853890.2021.2004319 (PMC8805968; doi:10.1080/07853890.2021.2004319)

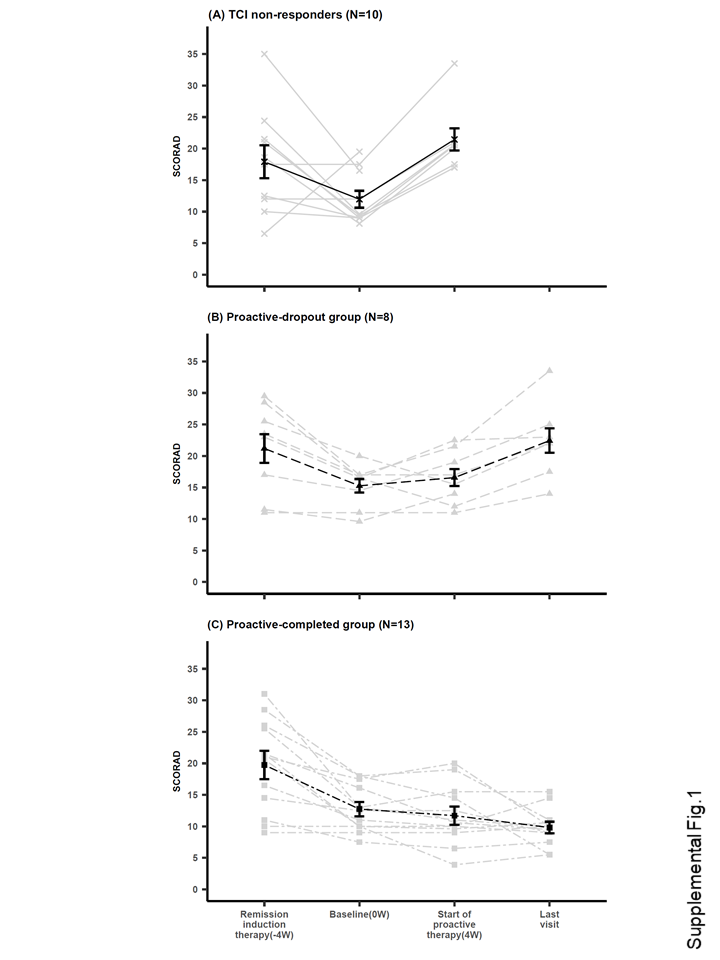

Supplement: Supplemental Material [file IANN_A_2004319_SM9574.zip › Supplemental files/Supplemental Figure1 revised.tif]

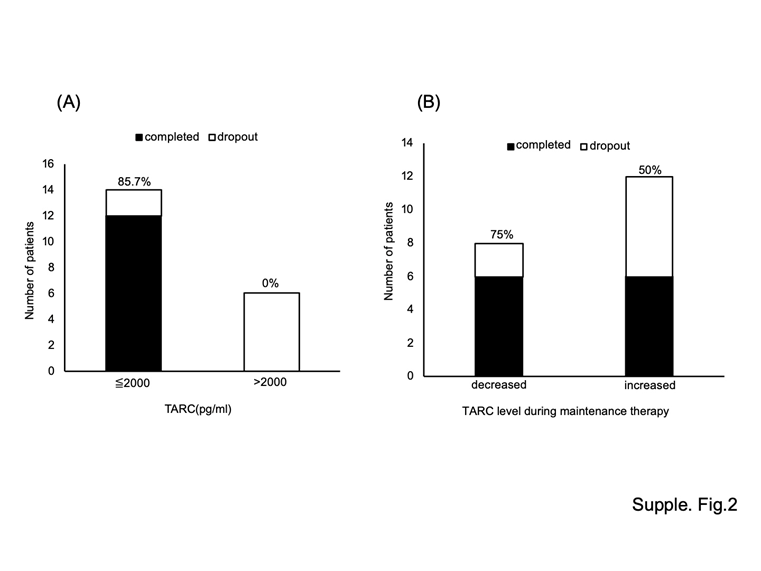

Supplement: Supplemental Material [file IANN_A_2004319_SM9574.zip › Supplemental files/Supplemental Figure2.tif]
